# Public Health Policy Based on “Made-In-Brazil” Science: A Challenge for the Arquivos Brasileiros de Cardiologia

**DOI:** 10.5935/abc.20150120

**Published:** 2015-09

**Authors:** Jussiely Cunha Oliveira, José Augusto Barreto-Filho

**Affiliations:** 1 Núcleo de Pós-graduação em Medicina - Universidade Federal de Sergipe, Aracaju, SE – Brazil; 2 Faculdade Estácio/Sergipe, Aracaju, SE – Brazil; 3 Divisão de Cardiologia – Hospital Universitário – Universidade Federal de Sergipe, Aracaju, SE – Brazil; 4 Clínica e Hospital São Lucas, Aracaju, SE – Brazil

**Keywords:** Cardiovascular Disease, Policy, Outcome Assessment (Health Care), Constitution and Bylaws, Unified Health System

To increase healthcare quality in a sustainable and equitable fashion is a major challenge in the contemporary world. Country governments need to continuously search for creative and intelligent solutions to align in a balanced way the multiple health subsystems with the needs and expectations of the patient and community, and such solutions have to be viable and sustainable. Developed countries use science to assess the quality of the health care provided and to generate information to improve the outcomes^1^. Science-based public policies are the current paradigm in those countries. In Brazil, however, the ideological viewpoint seems to prevail.

"Outcomes research", "health service research" and "implementation science" comprise a significant part of the themes related to the scientific study of health care quality and its relation to the health system^1^, but still lack disclosure and institutional incentives to thrive in Brazil. Those areas are usually devoted to systematically and methodically assess various aspects related to the structuration of health systems, their outcomes for the patient and community, physician-patient relationship, the ways such outcomes can be improved, and how innovations should be implemented. Out of the three, the "outcomes research" stands out, because it investigates the outcomes of health care focused by the perspective that directly interests patients and society^1^.

## Scrutinizing the quality of the Brazilian health system

Since the Brazilian 1998 Federal Constitution (Constitution of the Citizen) was signed and the Brazilian National Unified Health Care System (SUS) was created in 1990, the right to integral, universal, equalitarian and free access to health services of quality has been granted to all Brazilians. The State would have to finance, provide and operate the infrastructure necessary to fulfill the Law 8.080/1990. The constitutional recognition that health is a universal right was a substantial ideological advance. On paper, the public health model idealized for Brazil has become an example to the world. Approximately 75% of Brazilians are estimated to depend on the SUS for health care, while the remaining Brazilians have private complementary coverage.

If the objective is to generate relevant information to guide decision making regarding public policies on health, we should systematically dedicate ourselves to scientifically assess the health care outcomes of SUS. However, after 25 years from SUS implantation, there is little scientific evidence, especially that representing the continental country Brazil is, on the final health care outcomes of SUS. For example, representative data on post-acute myocardial infarction mortality, reperfusion treatment rate or hospital readmission rate in 30 days are not available. Moreover, little is known about the disparities of the health care outcomes between the SUS and the private health care system.

The consolidation and construction of an equitable, safe, responsive, accessible and efficient health system require the careful and scientific examination of the SUS and the Brazilian complementary health system at national level. The recent initiative of the Brazilian Society of Cardiology encouraging national registries, although still timid in transforming Brazilian public health, should be praised^2^.

DATASUS could play a role in surveilling the quality of the health care outcomes of the major Brazilian health problems. However, the unsatisfactory quality of data input and the lack of studies nationally validating DATASUS as a reliable data bank are usually limiting factors to its scientific use.

In the United States, administrative data from Medicare have been used in several studies on monitoring and surveillance of health care macro-indicators in the major cardiovascular pathologies^3-5^, significantly helping knowing the outcomes of the health care provided to North-Americans aged 65 years and older.

## Health care outcomes as a scientific theme

The concept of the imperative need to constantly assess the real-world health care outcomes to continuously improve the health system has been crystallized in the end-result idea by Ernst Codman (1910)^6^. According to Codman, the end-result idea requires the results to be constantly assessed and possible solutions to improve them to be constantly considered.

The theoretical basis of "outcomes research" was later refined by Donabedian, who has proposed a conceptual model^7^ in which the quality of the health system could be inferred by approaching the following three domains: structure, process and outcomes. The "outcome" domain is the one that best captures the quality of the health care that interests patients and society.

In 1998, the term "outcomes research" entered the scientific terminology in a classical publication in the journal *Science*. As defined by Clancy and Eisenberg, "outcomes research" investigates the effects of medical interventions and policies on the outcomes that directly interest individuals and society^8^.

Ten years later, when the American Heart Association launched the journal *Circulation Cardiovascular Quality and Outcomes*, edited by Krumholz and associates, "outcomes research" was definitely recognized as an important area of cardiovascular investigation, endorsing the emergent field of biomedical research^9^.

## Examples of studies on the quality of cardiological care in Arquivos Brasileiros de Cardiologia

The journal *Arquivos Brasileiros de Cardiologia* is the major vehicle of Brazilian cardiology and represents the Brazilian Society of Cardiology. We reviewed original articles published in the *Arquivos Brasileiros de Cardiologia* in the last two years on quality of health care, aiming at providing examples of studies that could contribute to and impact on the Brazilian health care outcomes.

To make our investigation more comprehensive, we subdivided the theme "quality of health care" into some subthemes that directly interest outcomes research and others that specifically interest Brazil, such as the focus on SUS^1^(Table 1). We intended to assess neither the quality of the study published nor its potential impact on generating guideline recommendations. Some studies served more than one category.

**Table 1:** Original articles on quality of care in Arquivos Brasileiros de Cardiologia

	Examples of studies*
Safety	*Eficácia e Segurança de Stents Eluidores de Drogas no Mundo Real: Acompanhamento de 8 Anos^10^*
Temporal line of care, access and responsiveness of the system	*Implantação da Linha de Cuidado do Infarto Agudo do Miocárdio no Município de Belo Horizonte*^11^
	*Efetividade de um Protocolo Assistencial para Redução do Tempo Porta-Balão da Angioplastia Primária^12^*
Variability in health care practice	None
Effectiveness	*Estratégia Antitrombótica nos Três Meses Iniciais após Implante de Bioprótese Valvar Cardíaca^13^*
Cost	*Itinerário de Investigação do Paciente Coronariano do SUS em Curitiba, São Paulo e Incor - Estudo IMPACT^14^*
Disparity	*Evolução de Indicadores Socioeconómicos e da Mortalidade Cardiovascular em três Estados do Brasil^15^*
Patient-centered care /autonomy / shared decision making	None
Institutional results	*Experiência Inicial de Dois Centros Nacionais no Implante de Prótese Aórtica Transcateter^16^*
Registries on specific diseases	*Registro Brasileiro das Síndromes Coronárias Agudas (ACCEPTf*
	*Estudo BREATHE -1 Registro Brasileiro de Insuficiência Cardíaca^17^*
	*Comportamento da Síndrome Coronariana Aguda. Resultados de um Registro Brasileiro^18^*
Focus on SUS	*Itinerário de Investigação do Paciente Coronariano do SUS em Curitiba, São Paulo e Incor - Estudo IMPACT^14^*

Although no systematic quantitative assessment was performed, we identified a notorious scarcity of investigation directly approaching the theme "quality of the health care" provided by the Brazilian health system.

## Challenges and opportunities

Our search, restricted to the *Arquivos Brasileiros de Cardiologia*, suggests that Brazil needs to increase its scientific production capable of guiding public policies in the cardiovascular setting, where the use of imported science has critical limitation and can bias decision making. Systematically knowing the Brazilian health care outcomes is essential to elaborate and prioritize the agenda of regional and national public policies.

We have a long way to go and at least the following two very well-defined challenges to face if we bet on science to support decision making regarding public policies on health: to produce high-level national science representing the quality of Brazilian health care; and to convince federal, state and municipal authorities that science is a fundamental tool to guide decision making on the implantation of public policies.

That requires substantial investment: 1) in the intellectual formation of specialized researchers; 2) in improving the quality of DATASUS as a data bank for research in all Brazilian states; 3) in the creation, structuration and consolidation of cooperative research groups; and 4) in the continuous encouragement of the national scientific production.

For the Brazilian cardiovascular scientific community, especially younger researchers, this gap in the Brazilian science can represent a great opportunity to embrace a research line that can substantially impact on and benefit Brazil and Brazilians.
